# Can Local Geographically Restricted Measurements Be Used to Recover Missing Geo-Spatial Data?

**DOI:** 10.3390/s21103507

**Published:** 2021-05-18

**Authors:** Hrvoje Kalinić, Zvonimir Bilokapić, Frano Matić

**Affiliations:** 1 Department of Informatics, Faculty of Science, University of Split, 21000 Split, Croatia; zvonimir.bilokapic@ericsson.com; 2 Institute of Oceanography and Fisheries, Šetalište I. Meštrovića 63, 21000 Split, Croatia; fmatic@izor.hr

**Keywords:** data reconstruction, machine learning, neural networks, missing data, spatio/temporal resolution, interpolation, reanalisys

## Abstract

The experiments conducted on the wind data provided by the European Centre for Medium-range Weather Forecasts show that 1% of the data is sufficient to reconstruct the other 99% with an average amplitude error of less than 0.5 m/s and an average angular error of less than 5 degrees. In a nutshell, our method provides an approach where a portion of the data is used as a proxy to estimate the measurements over the entire domain based only on a few measurements. In our study, we compare several machine learning techniques, namely: linear regression, K-nearest neighbours, decision trees and a neural network, and investigate the impact of sensor placement on the quality of the reconstruction. While methods provide comparable results the results show that sensor placement plays an important role. Thus, we propose that intelligent location selection for sensor placement can be done using k-means, and show that this indeed leads to increase in accuracy as compared to random sensor placement.

## 1. Introduction

Some measuring endeavors have a long history and provide valuable information [1,2,3,4]. Others include just scarce information that provides important glimpses into a history that would otherwise be undocumented [5,6]. In either case, they reflect the general fact that there is usually a lot of missing or unavailable data. Usually this happens due to unavailable resources [1,7,8]. However, even today, when there are many more resources available for data acquisition and data storage, and everything is happening at a faster pace, it is difficult to avoid data gaps. Sometimes the intrinsic physical limitations of the measurement endeavor do not allow data to be acquired with better spatial or temporal resolution [7,9] but often there is a trade-off between the resources spent on a measuring endeavor and the value derived from it. Thus, we often pursue a way to optimize the measuring endeavor in the sense of trying to maximize the value extracted from the measuring instruments while minimizing the resources spent on the measuring endeavor. It is often this mismatch that ensures the sustainability of an endeavor. Thus, if advanced machine learning can be employed to accurately estimate the data at certain locations, it would reduce the overall cost and encourage further measurement. Since we are interested in using certain measurements as proxies to estimate the data at locations where there are no measurements, a particularly interesting question is whether the location for such proxies can be intelligently estimated.

The geosciences are particularly well suited to the study of these questions because these problems arise naturally. Historically, many of these problems were due to hardware limitations or unavailable resources. Therefore, some of these problems have already been addressed in the literature. For example, meteorological time series are often incomplete at certain locations and times. It is not uncommon for time series to be created by merging data from multiple sources. A number of data imputation and interpolation techniques have also been developed, such as regression-based methods, kriging, and inverse distance weighting (e.g., refs. [10,11,12,13,14]). In oceanography, measurement stations, such as oceanographic buoys or radar measurements of sea surface temperature and surface current HF, are sparsely scattered in space and time. Self-organizing maps have been used to overcome the poor temporal resolution and fill in the missing data, validate the data, find the outliers and identify different climate regimes [8,15,16,17,18]. In satellite data, it is not uncommon to find situations where the spatial resolution of the acquired data is limited because often a large number of values are missing due to clouds, shadows and other atmospheric conditions. To address this problem, some studies have used a neural network model [19,20,21] or inpainting [22,23].

However, even today it is not too difficult to find situations where the spatial resolution of the acquired data is limited or where the data have poor temporal resolution. And this will continue to be the case regardless of how reliable the hardware we get will be. An interesting example of the first case is oceanographic monitoring stations that are fixed and scattered sparsely in space. While such stations (at least those on the coast) usually do have good temporal resolution, the spatial resolution of the data they collect is poor. An exemplar of the second case in the geosciences is satellite data. Data originating from earth observation satellites may have good spatial resolution and no occlusions (under perfect conditions), but their temporal resolution is limited by the time it takes them to orbit the Earth and observe the same geographic location [7,24,25]. Thus, while the use of satellites for data collection results in greater spatial coverage, the temporal resolution of such data may be inadequate. Therefore, one might still prefer to use the local data (with good temporal resolution) to qualitatively recover measurements from a larger geographic area. Ideally, this would cover the same area as covered by satellites, but also for a period when satellite data were not available. Of course, the quality of the reconstruction will depend on the quality of the data collected. The theoretical foundations for reconstruction of a signal from sparse data can be found in compressed sensing theory [26,27,28,29]. Furthermore, there is the question of whether there are particular locations from which are more suitable to acquire data from in order to obtain a better reconstruction. This problem appeared in several occasions and can usually be formulated as a selection problem or an optimization problem. Whether we pose the problem in terms of sensor (site) selection or optimal sensor placement, we can find a number of proposed solutions [30,31,32,33,34].

In order to find the answers to these kinds of questions, we set up the following experiment. First, we select a set of machine learning models to be used in a supervised learning setup. Specifically, these are: linear regression, k-nearest neighbors, decision trees, and a neural network. We then use available data to learn each of the selected models. We conducted several experiment by using different portion of data—ranging from 50% to 1%—to investigate how much data is needed for a good reconstruction. We also investigated how different instrument placement strategies would affect reconstruction accuracy. In each experiment, we compared multiple machine learning models.

In the following section, we (a) explain the data we use and how we define and measure the quality of the reconstruction; (b) describe four supervised learning techniques used as reconstruction methods; and (c) describe an unsupervised learning technique that can be utilized as an unbiased strategy for optimal site selection for the measurement stations. We then describe the experiment and present the results, which show how good reconstruction is possible even when only a small number of physical measurements are available. Furthermore, the results highlight the importance of the optimal sensor site selection strategy to achieve better reconstruction accuracy. We discuss this in more detail in the last section of the paper.

## 2. Materials and Methods

In this section we describe the data and the four supervised methods that are utilized for a data reconstruction problem at hand, as well as an unsupervised method, which is proposed to be used to identify optimal sites for sensor placement. Supervised methods implemented in our experiments appear in the following order: (a) linear regression, (b) K-nearest neighbors, (c) extra trees and (d) neural network. As an unsupervised method for an intelligent site selection the K-means clustering is used.

### 2.1. Data

**Figure 1:**
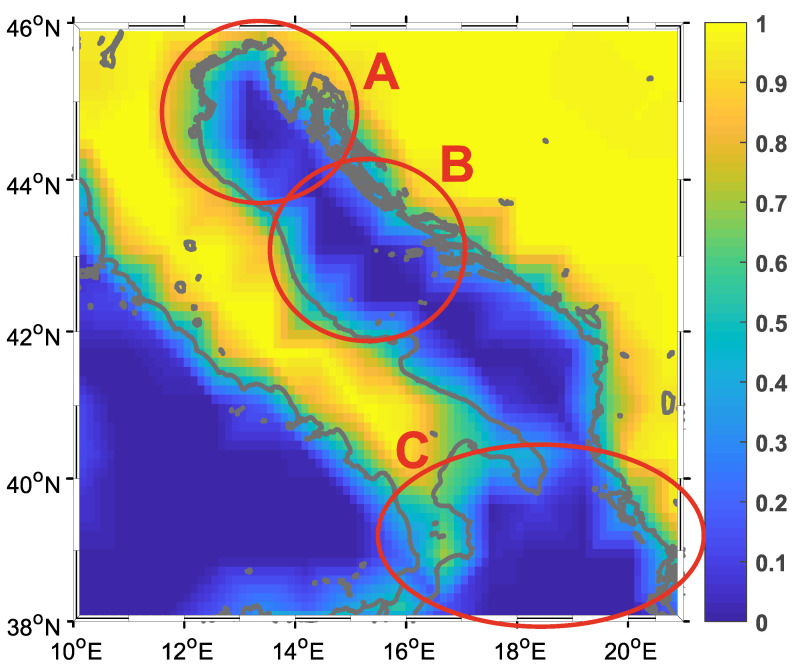
Adriatic sea—the geographic area covered in the study with marked selected zones: (**A**) North Adriatic, (**B**) Middle Adriatic and (**C**) Ionian Sea. Color bar shows wet point index defined by the ERAInterim land-sea mask.

**Figure 2:**
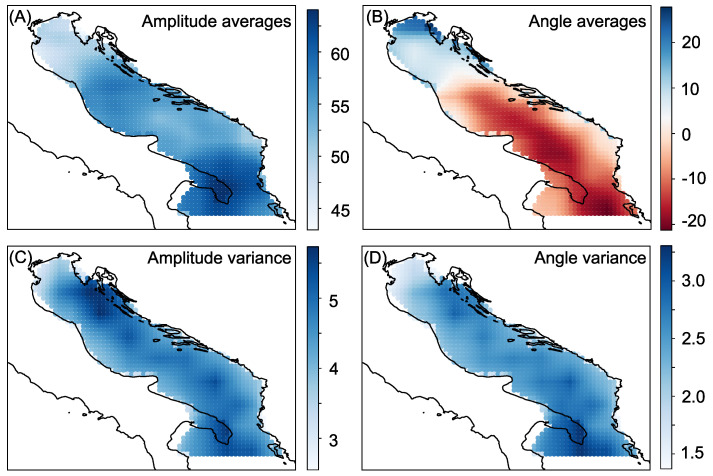
Adriatic sea—Panels show: (**A**) Amplitude average. (**B**) Angle average. (**C**) Amplitude variance. (**D**) Angle variance.

Data used in the study are from the European Center for Medium-range Weather Forecasts (ECMWF). ECMWF uses the ERA Interim reanalysis model and the data are available as part of the Copernicus Climate Change Service information [35]. The larger geographic area of this study is depicted in Figure 1. The color information in Figure 1 indicates whether the particular point in the reanalysis model is associated with land (dry point) or sea (wet point). In our study, we opted to define wet points as those below the threshold of 0.5, thus defining the land-sea mask. We used this information to extract the wind data at 10 m height over the Adriatic Sea and Northern Ionian Sea. The spatial distribution of the data, together with the data variability, is shown in Figure 2

The horizontal resolution of the wind data vector is 0.125° latitude and 0.125° longitude with a time step of 6 h. The data set is organized as a 2210-by-54056 matrix. Each row contains spatial data for a particular time step. It is a vector constructed by concatenating information from two independent information (channels) from the sensor. Technically, these are two orthogonal components of the wind—the west-east and the south-north wind components—usually denoted by *u* and *v*, respectively. In our study, *u* and *v* are taken at 10 m height and are expressed in meters per second. Each column is a realization in time from 1981 to 2017 in chronological order, sampled with a time step of 6 h.

The Adriatic Sea was chosen as the test area because it has a diverse coastline with many islands and high variability of the wind vector in both space and time domain [36]. Three zones are marked in Figure 1. The first zone—labelled A—is Northern Adriatic, where the definition of wet points does not always follow the coastline. At the same time, this is the zone with the lowest angular variance and a specific wind type that often differs from the rest of Adriatic Sea [37,38]. The second zone—labelled B—is part of Middle Adriatic Sea. This zone is known to have the highest angular variability and it can be observed that the wet points are well defined (only a few of them are partially wet). The third zone—labelled C—includes a part of Ionian Sea, known to have a relatively different wind regime, that is, a weak correlation with the wind in the rest of the studied area. Moreover, this area contains some partially wet points declared as “wet” by the land-sea mask.

**Figure 3:**
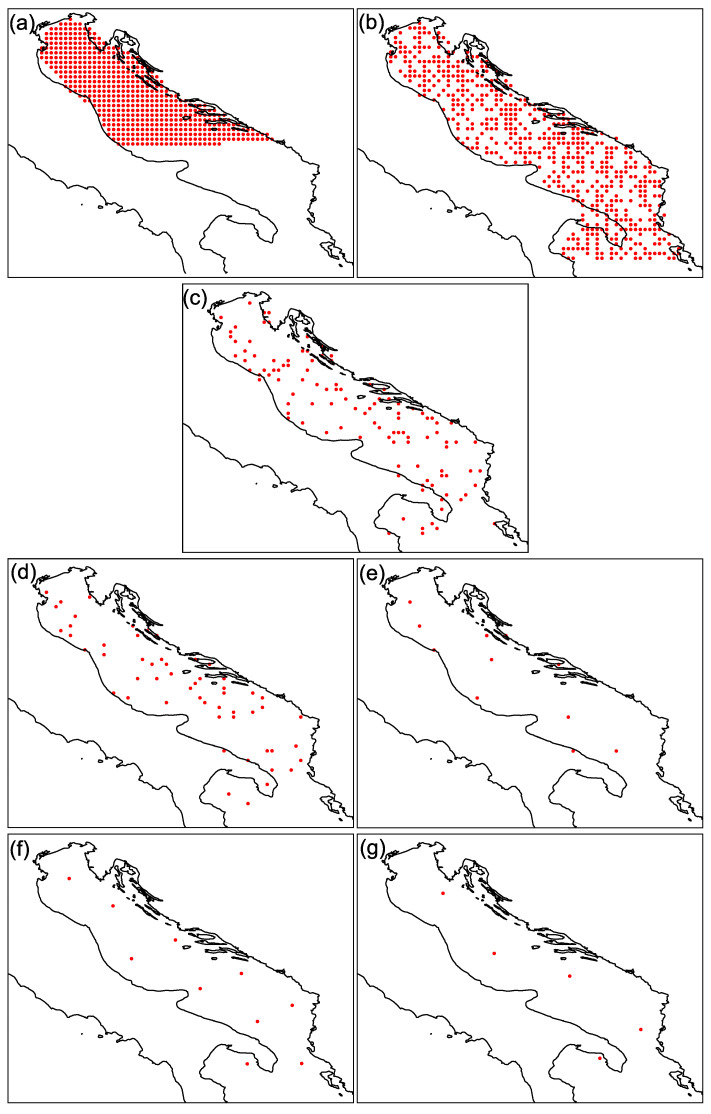
Sampling scenarios used in experiments (**a**–**g**): (**a**) 50% samples selected in successive order. (**b**) 50% samples selected randomly. (**c**) 10% samples selected randomly. (**d**) 5% samples selected randomly. (**e**) 1% samples selected randomly. (**f**) 10 samples selected by the k-means algorithm. (**g**) 5 samples selected by the k-means algorithm.

The locations from which the data were acquired are depicted in Figure 2. Different panels show either average values or standard deviation. The points in panels (A) and (B) of Figure 2 colour-code the average amplitude at that particular location and the average angle. Similarly, panels (C) and (D) depicts the variability of the data by colour-coding the variances of amplitude and angle at each point in the geographic area covered in the study. In Figure 2B, a characteristic wind pattern can be observed over the Adriatic Sea—wind from SE in the southern part and from NE in the northern part of Adriatic Sea [36]. All points shown in the figure are wet points and the discrepancy between the coastline and the wet point definition is due to the land-sea mask definition and the reanalysis model resolution. A portion of the data would be sampled from these points to simulate sensor placement, as explained in the Experimental Setup subsection and depicted in Figure 3.

### 2.2. Linear Regression

Linear regression (LR) is an old and simple supervised learning method usually used for predicting data. It assumes a relationship between the observed variable (*y*) and a set of *n* independent variables (*x*):

(1) $$\hat{y_{i}}=\beta_{0}+\beta_{1}x_{i,1}+\ldots +\beta_{n}x_{i,n},$$

The training data are used to estimate the coefficients β, and the resulting regression model is used to predict the future value of *y* based on *x*.

In general, a regression problem can be viewed as a problem of fitting data to a model. The least squares criterion for goodness of fit is by far the most common approach. Accordingly, for a regression model to be a good fit, the cumulative error across all sample data points and a model must be minimal:

(2) $$err_{reg}=\sum_{i}y_{i}-\hat{y_{i}}.$$

This can be formulated as an optimization problem (see Neural Networks subsection), but there is an analytical solution that is usually used. Moreover, linear regression is not to be confused with a PCA approach (Principle Component Analysis), which minimizes the orthogonal projection error on a subspace plane. It can be shown that minimizing the orthogonal projection error and maximizing the variance are equivalent optimization problems, so the optimization problem is usually formulated in the form:

(3) $$\arg\max_{||b||=1}\;\{b^{T}\sum_{i=1}^{k}x_{i}x_{i}^{T}b\}=\arg\max_{||b||=1}\;\{b^{T}X^{T}\mathrm{Xb}\},$$

where b contains the coefficients (or mappings) of the vector $x_{i}$ onto the new orthogonal space and X is just a matrix notation. Since PCA does not distinguish between observed and independent variables and treats all data the same, PCA falls into the category of unsupervised learning. In addition, linear regression is not to be confused with linear interpolation which requires the interpolation to pass through all the data points. Linear regression does not have this requirement, although it can be used to interpolate the data just as it can be used to predict future values.

### 2.3. K-Nearest Neighbors

K-nearest neighbors (KNN) is a technique commonly used in a classification problem where one tries to estimate the class (let us denote it by *y*) of an unknown vector *x* based on its neighborhood. In general, the neighborhood can be specified as a parameter ϵ—defining the distance from *x*—or as an integer *N*—defining the number of neighbors around *x* [39]. In either case, the neighbors are used to estimate the value of *y*, and usually the neighbors are defined using Euclidean distance as a metric. As the name implies, if a KNN model is used, *N* is the only parameter other than *x* that is passed to a model to estimate *y*. This also means that ϵ varies for different *x*s, depending on the distribution of data points.

In a classification scenario, KNN estimates the label *y* based on *x* like a voting machine—i.e., the majority class wins. Note that the label is a discrete value. If instead of a discrete value of *y*, the continuous values of *y* are preferred, KNN regression can be performed. When KNN is used as a regression, the default case is that all neighbors contribute equally. This can give unfair weight to points further away from *x*, but is considered more robust as it is less affected by outliers. Alternatively, not all neighbors need to contribute equally, but their contribution can be weighted according to their distance from *x*. In such a scenario, KNN can force an adjustment by the points from the training samples, resulting in a lower error on the training set, but not necessarily a lower error on the test set.

In a regression setting, KNN can be observed as a variable bandwidth kernel-based estimator. The estimated value *v* is then calculated as:

(4) $$v=\sum_{i=1}^{n}w_{i}\cdot c_{i},$$

where $w_{i}$ is the weighting factor and $c_{i}$ is the contribution of this neighbor $x_{i}$ to the value at position *x*. As mentioned earlier, $w_{i}$ can be proportional to the distance from $x_{i}$ to *x*, or otherwise be constant for all $x_{i}$, in which case it is inversely proportional to *k* and the size/radius of the neighborhood (i.e., 1/f(k,r)). The contribution from each $x_{i}$ is computed as a kernel function:

(5) $$c_{i}=K(\frac{x-x_{i}}{r}),$$

where *r* denotes the width of a kernel, and *K* is a kernel function, which can be any precomputed metric function, or in a simplistic case an Euclidean distance.

### 2.4. Extra Trees

Extra trees (ET) is a shorthand for extremely randomized trees [40], which is a meta-algorithm that utilizes multiple decision trees and an ensemble method to estimate a value. A decision tree is an algorithm usually used for a classification for which we may say it is non-parametric and easily interpreted. The algorithm uses no parameters (apart from data samples) and produces a result that can be easily interpreted as a set of if-then-else statements. We may see that the partition of the output space in this way may lead to a result with high quantization error. As a workaround for this problem one might propose to grow multiple trees and ensemble the results by averaging the values. This would increase the accuracy and as a side-effect introduce a regularization in the algorithm that controls the overfitting. On the other hand, this would make the model harder to interpret. This approach is the essence of the algorithm dubbed Extra Trees. One last part that is necessary to accomplish is to assure that the multiple trees that are randomly grown from the same sample points do not produce the same decision tree. In order for algorithm not to grow the same tree for the same data, a randomization may be induced by restricting the number of features used in a tree to a subset of the features, or by using the subsamples of data.

### 2.5. Neural Networks

Neural networks (NN) are powerful nonlinear methods whose power lies in the vast number of neurons organized in layers in which the information is processed in parallel. One neuron of the network may be observed as a weighted integrator of the form

(6) $$y=\sum_{i}w_{i}\cdot x_{i},$$

followed by nonlinear transformation of the data, that may be denoted as φ(.). So, in a vector notation this may be written as:

(7) $$y=\varphi (w^{T}\cdot x)$$

Compared to linear regression, the nonlinearity of a single neuron stems out. Note, however, that the neural network model has multiple occurrences of a neuron in a layer, and stacking multiple layers further contributes to the complexity (nonlinearity) of the model. If we want to pursue the similarity between regression and a neural network model further, we could say that the linear regression model fits the linear subspace hyperplane to the data by using the least squares criterion. Similarly, we could say that the neural network model fits the manifold (which could be viewed as the nonlinear equivalent of a subspace hyperplane) to the data. While an analytical solution exists for a regression problem, this is not the case when using neural networks. For this reason, to fit the neural network model to the data, one must define the loss function and an optimizer. The loss function is the objective that the model is trying to achieve. This can be, for example, the least squares criterion. The optimizer is a learning part of a neural network—an iterative process that ensures convergence. This can be, for example, the gradient descent algorithm.

### 2.6. K-Means

Unlike the previous methods, K-means is an unsupervised learning method. This means that it does not require any information from the supervisor. Compared to a classification problem that requires the data and labels (provided by the supervisor), K-means partitions the data based solely on the information provided from the data.

In our particular case, we are interested in such a method because we want to find an intelligent way to select the optimal location for data collection. We assume that data would naturally agglomerate in space, since spatial proximity is associated with correlation in virtually all natural processes. To identify these locations in space, we opt for a clustering approach that partitions the available data into non-overlapping clusters that arise naturally from the data. Then, the centers of these clusters can be selected as the optimal location to gather the data from.

A particular method that we utilize for this is a K-means. This method takes only one parameter, namely *K*—the number of clusters—and minimizes the within cluster variation (*W*()) for each cluster $C_{k}$, that is, $\sum_{k=1}^{K}W\left(C_{k}\right)$. The most straightforward approach to measure within cluster variation is to measure the Euclidean distance between all elements in a cluster (and normalize it to the number of elements in the cluster). However, this approach makes the optimization algorithm too complex since there are $K^{n}$ ways to partition a set of *n* elements to *K* clusters. Thus, the algorithm calculates centers of each cluster and measure how far apart the data is from the center. This approach is known to converge to local optimum, but in general provides good results. By utilizing this approach, the algorithm provides us with the *K*s centers—one for each of the clusters. This is precisely what we wanted in order to identify an optimal location for data acquisition. If required, a Voronoi tessellation may be utilized to identify the borders between clusters.

In a certain sense we may say that this is an intelligent approach that is less biased, as it requires and uses no additional external information—there are no labels, no supervisor, and only the information condensed in the data is used.

### 2.7. Definition of Error and Gold Standard

In order to measure the performance of each algorithm and compare the reconstruction accuracy as a function of the number of sensors used, we ought to specify how the error is calculated. At each site, two parameters are measured, namely *u* and *v*. One option would be to express the error as a term of each parameter (or channel), but since both channels measure wind, we chose to measure the error as the Euclidean distance between gold standard $X=(x_{1},x_{2},\ldots ,x_{n})$ and the reconstructed data $Y=(y_{1},y_{2},\ldots ,y_{n})$, i.e.:

(8) $$err_{X,Y}=\sqrt{{\left(x_{1}-y_{1}\right)}^{2}+{\left(x_{2}-y_{2}\right)}^{2}+\ldots {\left(x_{n}-y_{n}\right)}^{2}}.$$

Both $x_{i}$ and $y_{i}$ consist of two components—*u* and *v*. In the sequel, we will use the notation $\left(u_{i},v_{i}\right)$ to denote the gold standard and $\left({\hat{u}}_{i},{\hat{v}}_{i}\right)$ to denote the reconstructed data. This can be referred to as amplitude error and can be expressed using hat notation as follows:

(9) $$A_{err}=\sqrt{\sum_{i}^{n}{\left(u_{i}-{\hat{u}}_{i}\right)}^{2}+{\left(v_{i}-{\hat{v}}_{i}\right)}^{2}}.$$

Please note that we have access to all data points at Adriatic Sea from the beginning and that the missing values are simulated by omitting the available data. Therefore, the actual values can be used as a gold standard.

If we are interested in the angle between two vectors, we can use the cosine theorem:

(10) $$\phi_{err}=\arccos\frac{u\cdot \hat{u}+v\cdot \hat{v}}{\sqrt{u^{2}+v^{2}}\cdot \sqrt{{\hat{u}}^{2}}+{\hat{v}}^{2}}.$$

From these we calculate the average amplitude and phase error and the standard deviations of both amplitude and phase. The average amplitude error is then calculated across all locations in space (*S*) and all points in time (*T*)—and can be expressed as:

(11) $$avg\left(A_{err}\right)=\frac{1}{N_{S}}\frac{1}{N_{T}}\sum_{i\in S}\sum_{i\in T}A\left(err\right),$$

and the standard deviation as:

(12) $$std\left(A_{err}\right)=\sqrt{\frac{1}{N_{S}}\frac{1}{N_{T}}A{\left(err\right)}^{2}.}$$

The average angular error and standard deviation is calculated in a similar way.

## 3. Results

### 3.1. Experimental Setup

The data matrix contains *u* and *v* wind components of the data from across the Adriatic, and is organized as explained in previous section. Since we utilize learning models it is necessary to split the dataset to train and test set. This was done in the ratio 75:25, whereas the random 75% of realizations in time were used to train the models and the rest left for validation.

In order to utilize the supervised method, part of the training data is to be identified as an input data, while the other part is to be identified as a target data. In a supervised setting the target data is going to be used as if is provided from the supervisor, while the input data is used as if is provided from the on-site measuring instrument (sensor). The number and the location of the data points that act as an on-site measurements will vary across the different experiments (see Figure 3).

The goal of these experiments is to reconstruct the missing data. We learn to reconstruct the missing data from the input data (sensor) by providing the target data from the supervisor. To evaluate the quality of the reconstruction, we defined a measure of good reconstruction as a distance between the reconstructed data and the target data, which we used as a gold standard. To investigate the impact of the measuring instrument placement on the reconstruction error, we performed seven different experiments (denoted by (a)–(g)) in which we reduced the number of sensors or changed the strategy for their placement. First, we simulated a dense placement of instruments over the northern half of the Adriatic, that is, 50% of the data points were used as input data from sensors, all covering only the northern Adriatic. In the following experiments, we drew a random sample of data points over the entire Adriatic Sea to serve as data from field measurements. In this selection, 50%, 10%, 5% and 1% of the points were chosen as input data. Finally, in the last two experiments, 10 sensors (representing less than 1% of the data) and 5 sensors were selected using K-means clustering. The sampling scenarios for each experiment (a)–(g) are summarized in Table 1, and correspond to panels (a)–(g) of Figure 3.

In each experiment, reconstruction was performed using all the aforementioned machine learning models. Linear regression was used to estimate the coefficient based on the training data, which was then used to predict the missing data. K-nearest neighbors were used in a regression setting where each point in the neighborhood contributes equally to the missing value estimate. Additional trees were implemented by selecting random subsamples of data and then averaging the results obtained from multiple trees. Ten decision trees were grown prior to averaging. As a final method for reconstruction, a‘neural network with two hidden layers of size 500 and 250 was constructed. In the learning process, Root Mean Square Propagation (RMSprop) was used as the optimizer and mean square error as the loss function. In each experiment, the reconstruction error and its variance were measured for each of the machine learning algorithms used on the dataset.

### 3.2. Experimenal Results

The mean reconstruction error (ϵ) and its variance (σ) for each experiment are presented in Table 2. The letter denoting the experiment in the Table 2 corresponds to the equivalent sampling depicted in the panels of Figure 3.

When comparing experiments (a) and (b) from the Table 2, the importance of the sampling strategy stands out. Moreover, the error of the linear regression model from experiment (b) is virtually zero. This might be attributed to the true resolution of the ERA Interim model. A closer look at Figure 1 reveals this fact, as the transition between values is much coarser (this is perhaps most easily observed in region B) than the spatial distribution of the data would suggest. However, it is interesting to note two things: (i) linear regression performs well across all subsequent experiments and (ii) although the differences between the machine learning algorithms are not that large, it turns out that KNN and Extra Trees have the largest error across all experiments. Experiments (e) and (f) again fortify the conclusion that the selection of the optimal location for sensor placement significantly reduces the error and perhaps plays a more important role than the actual machine learning method used for reconstruction. A similar observation can be made when comparing experiments (e) and (g), as (e) uses twice as many sensors as (g). In the latter experiments, where a smaller number of sensors is available—that is, more data are reconstructed—neural networks outperform all other models.

**Figure 4:**
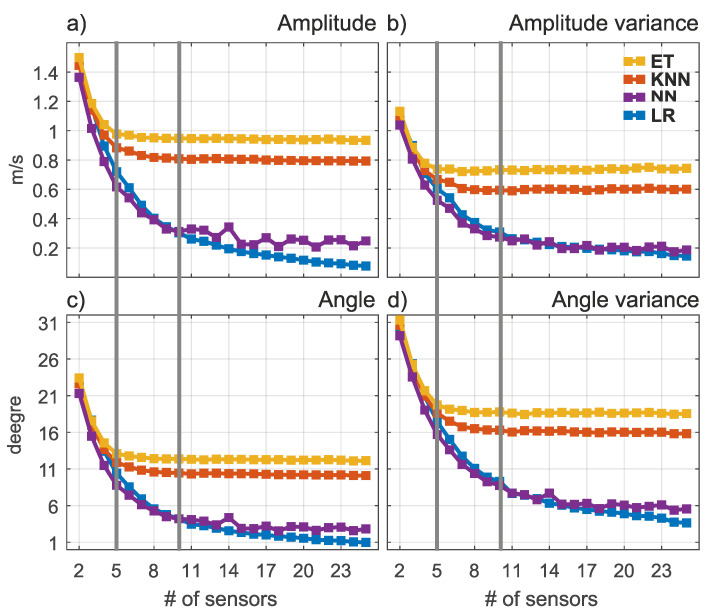
The average amplitude and angular error (subfigures **a**,**c**) and their variances (subfigures **b**,**d**) for different number of sensors. The gray line indicates experiments (f) and (g).

The excellent performance of the linear regression model in the experiments where the sensors densely cover the region of interest can be attributed in part to the data source. As can be seen in Figure 1 (region B), the intrinsic spatial resolution of the data can be coarser than the actual resolution available. Therefore, we are particularly interested in cases where a small number of sensors were used to reconstruct the missing information. Previous experiments indicated that when the number of sensors is small, sensor placement plays a more important role than the actual data reconstruction method. We used the intelligent sensor location selection method based on the k-means algorithm to select locations for different numbers of sensors ranging from 2 to 25. The results are shown in Figure 4.

Figure 4 shows the expected behavior. For all reconstruction methods, the error and variance decreases with a larger number of sensors, and this is consistent with the previous results—the gray line denotes the results of experiments (f) and (g) from the Table 2. However, it is interesting to note that KNN and Extra Trees have virtually no further gain when more than 10 sensors are used in the given domain. In fact, the results from Table 2 suggest that the gain in accuracy is possible but not very large for a much larger number of sensors used.

**Figure 5:**
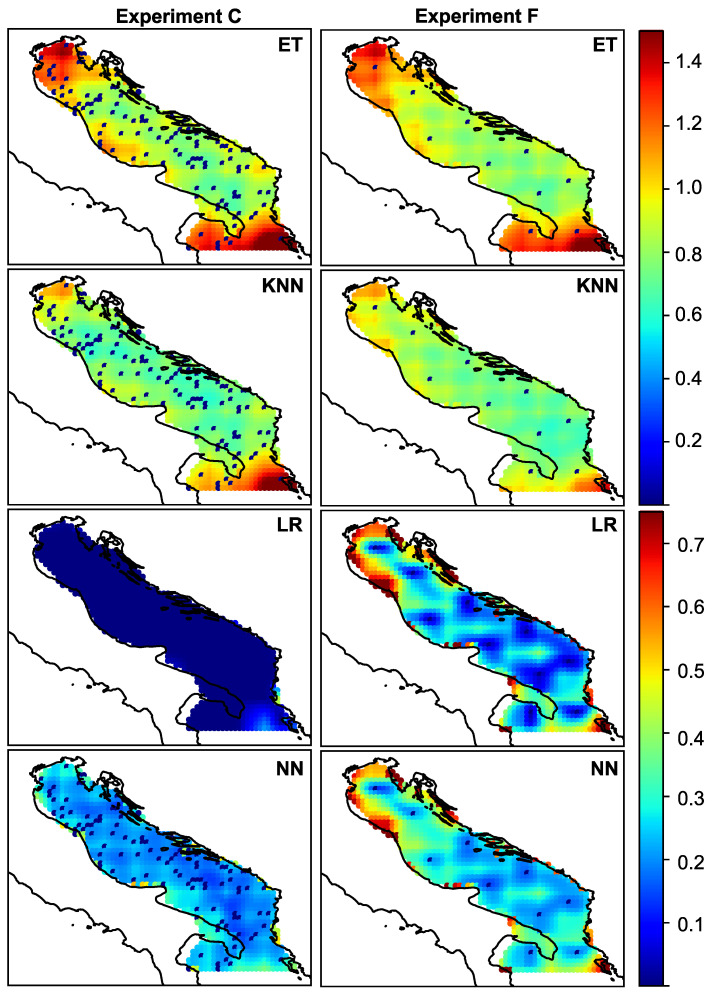
Spatial distribution of amplitude error for randomly distributed 110 sensors—experiment (c)—and for intelligent selection of the 10 sensors-experiment (f).

**Figure 6:**
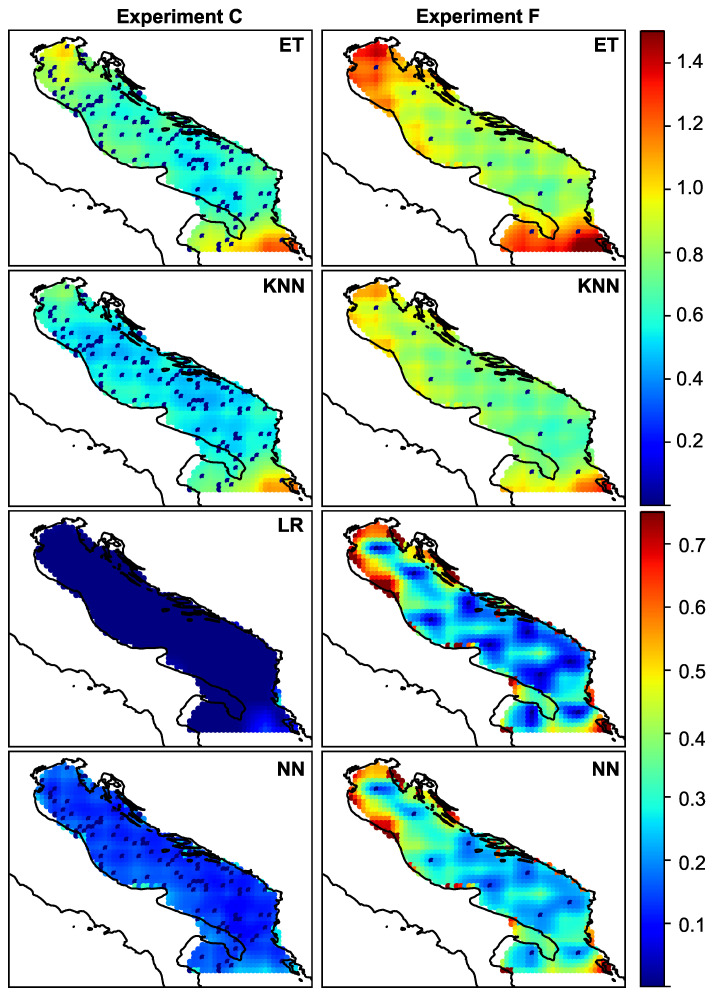
Spatial distribution of amplitude variance for randomly distributed 110 sensors—experiment (c)—and for intelligent selection of the 10 sensors experiment (f).

In all previous experiments, only the total error was observed. If we want to study the spatial distribution of the error, we should plot the amplitude and angular error for each experiment. The spatial distribution of amplitude and angular error is visualized in Figure 5 and Figure 6, respectively. The figures show the error for each of the ML models for experiments C and F, whose total error is given in Table 2.

From the figures, we can observe the spatial distribution of the error and discuss the peculiarities. It is to be expected that the error is more or less uniformly distributed, and that the closer one gets to the sensor, the smaller the error. This is especially noticeable at first glance. However, it can be seen that the error increases as one approaches the edges of the region of interest. Regardless of the density of the sensors, the northern and southern parts of the Adriatic have large reconstruction errors when KNN and Extra Trees are used for reconstruction. The variance follows this behavior. In experiment (f), this spatial pattern of error and variance can be observed for all ML models. This corresponds to regions A and C as indicated in Figure 1, and as mentioned earlier, these regions are known to have specific wind types and different wind regimes. As can be seen from the figures, the choice of model for data reconstruction or an increase in the number of sensors can further reduce the error in these regions. If we reduce (or neglect) this type of error, we can see that there is another type of error, namely the reconstruction error that occurs in the coastal zone. The points at the coastal zone are shown as “partially” wet in the land-sea mask. In this zone, the wind vector changes from continental to open sea regime, it changes from local (coastal) to global (open sea) meteorological processes. As a result, the process at this particular point is less correlated with processes at neighbouring points. This could be particularly important for future work, as most sensors in real scenarios are located in the coastal zone and could be a poor choice for optimal sensor locations when sensor information is used to reconstruct wind data.

Looking at the spatial distribution of error and variance shown in Figure 5 and Figure 6 for the experiment with the lower number of sensors (f), we can see that the reconstruction error of the neural network and linear regression model is less than 0.1–0.2 m/s for most of the observed region.

## 4. Discussion and Conclusions

The aim of this study was to investigate the possibility of using part of the synoptic data to reconstruct the overall picture of a synoptic situation. Wind data from a broader Adriatic region were used as a case study, and several machine learning techniques were applied to the data. The overall results show that the average amplitude error is an order of magnitude smaller than the mean and is comparable to the uncertainty of a hardware sensor measurement. Furthermore, a relatively small amount of data is required to achieve good amplitude reconstruction, that is, only a few percent of the data is sufficient to ensure that the average amplitude error is an order of magnitude smaller than the average amplitude. This is true for all machine learning methods used here. In fact, it can be seen from the results that the different implementations of machine learning perform comparably in the task of data reconstruction from this dataset. The results suggest that the data sampling—that is, the selection of the location from which the input data comes—plays a more important role than the particular machine learning algorithm. The different proportion of data—from 50% to 1%—is used as input data to investigate how much data is needed for a good reconstruction. In addition, different input data selection strategies are used to investigate how different instrument placement strategies would affect the reconstruction accuracy. Based on the fact that changing the input data selection strategy leads to a significant change in the overall reconstruction performance, we conclude that it is more important for an on-site implementation to have an intelligent way to select the locations where measurements are collected than an intelligent algorithm for data reconstruction. Based on the results of this study, which was conducted using only wind data, we can conclude that local, geographically restricted wind measurements can be used to recover missing wind data, and that this is a good indication that, in general, local, geographically restricted measurements can be used to recover missing geospatial data from a larger geographic area.

## Figures and Tables

**Table 1:** Sampling scenarios used in experiments (a) to (g).

Experiment	Sampling Model	# of Sensors
(a)	pre order	50% (1105 sensors)
(b)	random	50% (1105 sensors)
(c)	random	10% (110 sensors)
(d)	random	5% (55 sensors)
(e)	random	1% (10 sensors)
(f)	k-means	- 10 sensors
(g)	k-means	- 5 sensors

**Table 2:** Table containing mean reconstruction error (ϵ) and its variance (σ) for each of the machine learning model used in each experiment. The error is divided in amplitude and variance error.

Experiment		Linear		KNN		Extra		Neural	
		Regression				Trees		Network	
		ϵ	σ	ϵ	σ	ϵ	σ	ϵ	σ
(a)	Amplitude	0.52	1.00	0.91	1.42	0.76	1.23	0.73	1.17
	Angle	6.28	17.03	11.43	24.40	9.49	21.34	8.79	19.88
(b)	Amplitude	0.00	0.00	0.40	0.58	0.48	0.71	0.17	0.25
	Angle	0.00	0.00	5.16	12.33	6.24	14.63	2.00	5.12
(c)	Amplitude	0.00	0.03	0.74	0.64	0.87	0.77	0.20	0.16
	Angle	0.09	0.89	9.50	15.80	11.34	18.29	2.63	5.08
(d)	Amplitude	0.03	0.14	0.79	0.66	0.92	0.76	0.27	0.24
	Angle	0.40	2.72	10.20	16.47	11.89	18.55	3.17	6.32
(e)	Amplitude	0.52	0.68	0.94	0.81	1.02	0.84	0.55	0.59
	Angle	6.61	13.86	12.08	18.93	13.47	20.25	6.88	13.07
(f)	Amplitude	0.30	0.30	0.80	0.59	0.94	0.73	0.32	0.28
	Angle	4.20	9.30	10.44	16.29	12.37	18.76	4.31	8.95
(g)	Amplitude	0.72	0.60	0.88	0.66	0.97	0.73	0.61	0.52
	Angle	10.42	17.44	11.89	18.61	13.07	19.74	8.75	15.65

## Data Availability

Not applicable.

## Abbreviations

The following abbreviations are used in this manuscript:

LR	linear regression
KNN	K-nearest neighbours
ET	extra trees
NN	neural network
PCA	Proncipal Component Analysis
ECMWF	European Center for Medium-range Weather Forecasts
